# Which early life events or current environmental and lifestyle factors influence lung function in adolescents? – results from the GINIplus & LISAplus studies

**DOI:** 10.1186/s12931-017-0619-5

**Published:** 2017-07-12

**Authors:** Agnes Luzak, Elaine Fuertes, Claudia Flexeder, Marie Standl, Andrea von Berg, Dietrich Berdel, Sibylle Koletzko, Joachim Heinrich, Dennis Nowak, Holger Schulz

**Affiliations:** 1 Helmholtz Zentrum München - German Research Center for Environmental Health, Institute of Epidemiology I, Ingolstädter Landstr. 1, 85764 Neuherberg, Germany; 2 ISGlobal, Centre for Research in Environmental Epidemiology (CREAL), Doctor Aiguader 88, 08003 Barcelona, Spain; 3 Universitat Pompeu Fabra (UPF), Plaça de la Mercè 10, 08002 Barcelona, Spain; 4 CIBER Epidemiología y Salud Pública (CIBERESP), Av. Monforte de Lemos, 3-5. Pabellón 11, 28029 Madrid, Spain; 5 Department of Pediatrics, Research Institute, Marien-Hospital Wesel, Pastor-Janßen-Str. 8-38, 46483 Wesel, Germany; 6 Dr von Hauner Children’s Hospital, Ludwig-Maximilians-University of Munich, Lindwurmstr. 4, 80337 Munich, Germany; 7 Comprehensive Pneumology Center Munich (CPC-M), Member of the German Center for Lung Research, Max-Lebsche-Platz 31, 81377 Munich, Germany; 8 Institute and Outpatient Clinic for Occupational, Social and Environmental Medicine, University Hospital of Munich (LMU), Ziemssenstr. 1, 80336 Munich, Germany

**Keywords:** Adolescence, Spirometry, Lung function, Determinants, Epidemiology

## Abstract

**Background:**

Various factors may affect lung function at different stages in life. Since investigations that simultaneously consider several factors are rare, we examined the relative importance of early life, current environmental/lifestyle factors and allergic diseases on lung function in 15-year-olds.

**Methods:**

Best subset selection was performed for linear regression models to investigate associations between 21 diverse early life events and current factors with spirometric parameters (forced vital capacity, forced expiratory volume in 1 s and maximal mid-expiratory flow (FEF_25–75_)) in 1326 participants of the German GINIplus and LISAplus birth cohorts. To reduce model complexity, one model for each spirometric parameter was replicated 1000 times in random subpopulations (*N* = 884). Only those factors that were included in >70% of the replication models were retained in the final analysis.

**Results:**

A higher peak weight velocity and early lung infections were the early life events prevalently associated with airflow limitation and FEF_25–75_. Current environmental/lifestyle factors at age 15 years and allergic diseases that were associated with lung function were: indoor second-hand smoke exposure, vitamin D concentration, body mass index (BMI) and asthma status. Sex and height captured the majority of the explained variance (>75%), followed by BMI (≤23.7%). The variance explained by early life events was comparatively low (median: 4.8%; range: 0.2–22.4%), but these events were consistently negatively associated with airway function.

**Conclusions:**

Although the explained variance was mainly captured by well-known factors included in lung function prediction equations, our findings indicate early life and current factors that should be considered in studies on lung health among adolescents.

**Electronic supplementary material:**

The online version of this article (doi:10.1186/s12931-017-0619-5) contains supplementary material, which is available to authorized users.

## Background

**Fig. 1:**
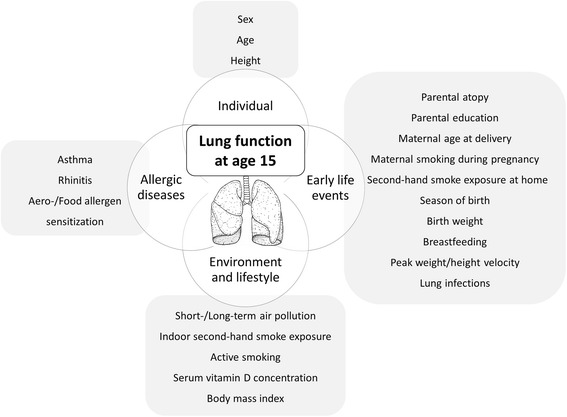
Covariates investigated for association with lung function in 15-year old adolescents (definitions presented in Table 1)

Lung development begins in early gestational age and continues until early childhood, while lung growth continues until 20–25 years, at which point a plateau in lung function is reached [1, 2]. Several factors have the potential to affect lung function during this process [1–4]. Adverse events in early life may influence lung function trajectories and lead to higher susceptibility to lung diseases, such as asthma or chronic obstructive lung disease [1, 5]. Recently published results among children with asthma underline that the impairment of lung function in childhood is a predictor of reduced lung-function growth and abnormal decline over time [4]. There is an increasing focus on the influence of lung function deviations in early childhood on later life respiratory morbidity [1, 4–7]. Numerous epidemiological studies have investigated factors that might influence lung function or that are associated with allergic respiratory diseases at different stages in life (Fig. 1). However, previous studies have mainly investigated only one factor or a few factors at specific periods of life, and most are focused on early life events [7–27]. In reality, it is likely that a complex framework of several factors determines an individual’s lung function throughout life [1, 2, 5]. Therefore, an approach that investigates the simultaneous effects of several factors might have the potential to identify which factors may be most influential at a certain stage of life. To date, only one epidemiological study reported on the effects of several factors (including early life events, socioeconomic status and environmental factors) on spirometric lung function between 6 and 16 years of age in Tunisian children [28]. Besides well-established factors (height, weight, sex and age), this study found that type of heating had the strongest effect on lung function in healthy Tunisian children.

In the current study, we used two large, well characterized, longitudinal German birth cohort studies to investigate associations between numerous early life events and current environmental and lifestyle factors, as well as allergic diseases, with lung function assessed by spirometry in adolescents aged 15 years. Our main purpose was to identify factors associated with spirometric measures of central and peripheral airway function in adolescents and further, to examine the relative importance of early life events compared to current environmental and lifestyle factors (Fig. 1).

## Methods

### Study population

We used data from two prospective German birth cohorts with each 15 years of follow-up; the German Infant study on the influence of Nutrition Intervention plus air pollution and genetics on allergy development (GINIplus) [29] and the study on Life-style related factors on the development of the Immune System and Allergies in East and West Germany plus the influence of traffic emissions and genetics (LISAplus) [30]. The inclusion criteria were the same for both cohorts, German families with a full-term newborn and birth weight of at least 2500 g were considered as eligible.

In GINIplus, 5991 neonates were recruited between 1995 and 1998 in the study centers Munich and Wesel and surrounding areas. Parents who’s newborns had at least one first degree family member with an atopic disease were asked to participate in the intervention study arm, which investigated the effect of three different hydrolyzed formulas on allergy development (*N* = 2252). All others were asked to participate in the observation study arm (*N* = 3739). At the 15-year follow-up, 1887 subjects participated in lung function measurements (50.4% were from the intervention arm).

In LISAplus, 3094 full-term children were recruited between 1997 and 1999 in the area of four German cities: Munich, Wesel, Leipzig and Bad Honnef. Given that air pollution concentrations (an important environmental factor considered in the analysis) were only available for Munich and Wesel in GINIplus, the population of LISAplus was restricted to these study areas, comprising 1812 subjects, of which 563 participated in lung function measurements at 15 years.

In GINIplus, parent-completed questionnaires were collected at birth, yearly from 1 to 4, and at 6, 10 and 15 years of age. In LISAplus, follow-ups were at birth, 0.5, 1, 1.5, 2, 4, 6, 10 and 15 years of age. The 15-year follow-up for both studies included a self-report questionnaire for the adolescents, lung function testing and blood sample collection. Further information about the cohorts’ design is described elsewhere [29–31]. Data from the LISAplus and GINIplus birth cohorts were pooled and are presented for the complete study population considering study group and study center as potential confounders in the analyses.

### Lung function measurements

Lung function measurements by spirometry were performed in line with ATS/ERS recommendations [32]. Participants were asked not to change their asthma medication prior to lung function testing. Among the analyzed participants, 83 had asthma. Of these, 89.2% reported asthma medication in the past 12 months. On the day of lung function testing, 7.2% reported the intake of short-acting beta agonists, 9.6% reported the intake of inhaled corticosteroids, and 18.1% reported the intake of both inhaled corticosteroids and beta adrenergic agonists, of which most (86.7%) consisted of long-acting beta agonists.

For the performance of spirometry assessments, the technicians were equally trained and the equipment used was the same in both study centers. Flow-volume curves were obtained using a pneumotachograph-type spirometer (EasyOne Worldspirometer, ndd, Zurich, Switzerland). Subjects performed at least three and up to eight trials per test. Trial results were visually inspected according to ATS/ERS acceptability criteria [32]. Indices of the best manoeuvre, defined as the test with the largest sum of the forced expiratory volume in one second (FEV_1_) and the forced vital capacity (FVC), were used in analyses. Further spirometric parameters obtained included the peak expiratory flow (PEF), the forced expiratory flow rates at 25% (FEF_25_), 50% (FEF_50_) and 75% (FEF_75_) of exhaled FVC and the mean flow rate between 25% and 75% of FVC (FEF_25–75_).

These parameters could be viewed as indicative of different lung regions or functions [33, 34]: lung volume (FVC), airways and lung volume (FEV_1_), airflow limitation (FEV_1_/FVC), flow rates for the larger conducting (PEF, FEF_25_) and peripheral airways (FEF_50_, FEF_75_ and FEF_25–75_). We focused our analyses and results primary on four spirometric parameters (FVC, FEV_1_, FEV_1_/FVC and FEF_25–75_) that represent lung volume and airway function. We also report if the associations for the primary parameters are supported by the results of the additional secondary parameters, which represent flow rates (PEF, FEF_25_, FEF_50_, FEF_75_). Results for the secondary parameters are presented in the additional file only.

### Definition of covariates

We selected factors for investigation based on a short review of the literature, including former GINIplus and LISAplus publications [8, 9, 35, 36], and after considering the number of participants with available data in our cohorts. An overview of investigated covariates is presented in Fig. 1. A detailed description was provided in Table 1. Investigated covariates were divided in early life events (e.g. parental atopy, maternal smoking during pregnancy, season of birth, birth weight), current environmental and lifestyle factors that were assessed at the 15-year follow-up (e.g. air pollution, indoor second-hand smoke exposure, BMI), and allergic diseases (e.g. asthma, rhinitis). Besides study specific variables (study group and center), sex, age and height, which are included in lung function prediction equations [37], were considered as basic covariates. The study population comprised only Caucasians, so ethnicity was not included as covariate.

**Table 1:** Definition of early life events, environmental and lifestyle factors, and allergic diseases at age 15

	Definition	Assessment
Early life events		
Parental atopy	positive if the mother or father had asthma, eczema or hay fever	asked at birth; questionnaire-based
Parental education	three categories based on the highest number of education years of either parent (high: >10 years; medium: 10 years; low: <10 years)	asked at birth; questionnaire-based
Maternal age at delivery	dichotomized in ≤31 years and >31 years (mean age served as cut-off)	asked at birth; questionnaire-based
Maternal smoking during pregnancy	yes vs no	asked at birth; questionnaire-based
Early second-hand smoke exposure at home	positive if parents reported at least once that the child was exposed to second-hand smoke at home	asked up to age 4 (at 4 months, 1 year (control arm only) and yearly at 2 to 4 years in GINIplus; half-yearly from birth to 2 years and at age 4 years for past 24 months in LISAplus); questionnaire-based
Season of birth	dichotomized (December to February (winter) versus other seasons)	Birth month derived from date of birth; questionnaire-based
Birth weight	continuous, grams	asked at birth in LISAplus and at 1 year in GINIplus; questionnaire-based
Breastfeeding	exclusive breastfeeding for at least four months	asked separately for 1–6 months at 1 year in GINIplus and at 6 months in LISAplus; questionnaire-based
Peak weight and peak height velocity	maximum of the first derivative of the individual weight or height gain curves obtained between birth and two years of age (calculated using nonlinear random effects models) [9]	weight and height measurements obtained during the children’s preventive medical check-ups to monitor growth
Early lower respiratory tract infections	doctor’s diagnosis of pneumonia or obstructive bronchitis within the first three years of life (hereon referred to as lung infections)	asked up to age 3 (yearly in GINIplus; half-yearly up to age 2 years and up to age 3 years asked at the 4 year follow-up in LISAplus); questionnaire-based
Environmental and lifestyle factors at age 15		
Short-term air pollution exposure	continuous, the average of the daily concentrations of NO_2_, PM_2.5_ mass and PM_10_ mass (μg/m^3^)	obtained for the seven days prior to lung function testing from monitoring sites near the centers of Munich and Wesel [8]
Long-term air pollution exposure	continuous, long-term concentrations of NO_2_, PM_2.5_ mass and PM_10_ mass (μg/m^3^)	estimated to each participant’s home address at birth, 10- and 15-years, respectively [8]
Regular indoor second-hand smoke exposure	positive if the adolescent reported indoor second-hand smoke exposure at least once a week	asked at age 15 years; one question in GINIplus, two questions for second-hand exposure: (1) at home and (2) in other locations in LISAplus (positive if regular exposure was reported in at least one question); questionnaire-based
Active smoking	yes vs no	asked at age 15 years, questionnaire-based
Vitamin D concentrations	continuous; serum 25-hydroxyvitamin D [25(OH)D] concentrations adjusted for seasonal variance using a generalized additive model (nmol/l) [36]	measured at age 15 years using Roche’s vitamin D total test (E170, Roche Diagnostics, Mannheim, Germany)
Body mass index	continuous, kilogram per square meter (kg/m^2^)	calculated using body height and weight obtained at lung function testing
Current allergic diseases		
Asthma	defined based on the Global Allergy and Asthma European Network (GA2LEN) definition [44]. Subjects were considered as currently having asthma if they responded positively to at least two of the three following questions: (1) Has a doctor diagnosed asthma in your child at the age 3 to 15 years? (2) Has your child taken asthma medication during the last 12 months? (3) Has your child had wheezing or whistling in the chest in the last 12 months?	parents were asked to provide yearly information on their child’s doctor diagnosed allergic diseases throughout childhood; information on current allergic symptoms and asthma medication in the last 12 months was derived from the last follow-up questionnaire at age 15 years.
Current allergic rhinitis	positive if one of the following questions was positive: (1) Has a doctor diagnosed hay fever (i.e. seasonal allergic rhinitis) in your child? (2) Has a doctor diagnosed perennial allergic rhinitis in your child?	asked separately for ages 11 to 15, at the 15-year follow-up;questionnaire-based
Sensitization to food or aeroallergens	food allergen mixture including egg white, codfish, cow milk, wheat flour, peanut, and soybean and aeroallergen mixture including cat, dog, mugwort, birch, timothy, rye, *Cladosporium herbarum*, and *Dermatophagoides pteronyssinus*; for both tests, a value >0.35 kU/L was considered positive	measured by serum specific Immunoglobulin E (IgE) using the ImmunoCAP Specific IgE system (Phadia GmbH, Freiburg, Germany) at age 15 years

GINIplus: German Infant study on the influence of Nutrition Intervention plus air pollution and genetics on allergy development; LISAplus: Life-style related factors on the development of the Immune System and Allergies in East and West Germany plus the influence of traffic emissions and genetics

### Statistical analysis

Differences between sexes and between participants included and excluded in this analysis were assessed using the t-test (normally distributed) or Wilcoxon rank-sum test (non-normally distributed) for continuous variables. The chi-square test was used for categorical variables.

Associations between study specific variables, early life events, environmental and lifestyle factors, and allergic diseases, with spirometric parameters were analyzed using linear regression. In order to determine the relevant factors, best subset selection was performed using the Mallows’ Cp statistic (Cp) as the model selection criterion. The Cp is based on least squares estimation and compares the precision and bias of a full model to models with a subset of all the independent variables, taking the number of predictors into consideration [38]. Best subset selection provides the model with the lowest Cp for all possible model sizes of the set of all potential independent variables. The model with the lowest Cp among all model sizes was chosen. Categorical variables were entered using dummy coding.

To strengthen the model selection approach, we applied a two-step process. First, we performed the selection in the total population. Second, to determine if the same variables would have been selected in a subpopulation or if any variables might have been selected by random, the model selection process was repeated 1000 times with two thirds (*N* = 884) of the population randomly selected for inclusion each time. The frequency of selecting a given variable in the regression models with the lowest Cp (even if the variable was not necessarily significant) was assessed and compared to the variables included in the models derived using the total population. To reduce model complexity, we focused only on variables that remained in >70% of the replication models for each particular spirometric parameter. Regression models including these selected variables (plus significant study specific variables) were rerun in the total population (*N* = 1326). Multicollinearity in the final models was assessed by the variance inflation factor (VIF), which is a measure of how much of the variance of an estimated regression coefficient is influenced by the correlation between independent variables. If correlations among variables exist, their relative importance, meaning the partial contribution to the total R^2^ of a regression model, is influenced by the order in which the variables are entered in a model. To adjust for possible correlations, we report the relative importance of each variable as the sequential R^2^ contribution [39]. The sequential R^2^ is corrected for the dependence on orderings by unweighted averaging of R^2^ contribution over all possible orderings. The reported results of the total sequential R^2^ per variable were normalized to sum up to 100% to facilitate comparability [39].

In a sensitivity analysis, current asthmatics were excluded from the final models.

Analyses were run in the statistical program R, version 3.2.0 [40]. *P*-values <0.05 were considered statistically significant.

## Results

### Study population

Valid lung function data and information on the investigated factors (Fig. 1) was available for 1326 subjects (63% from Munich, 51% male, mean age of 15.2 years; Table 2). Due to non-random loss to follow-up over the 15-year period, participants differed from the initial cohort, e.g. higher educated parents, more breastfeeding, lower BMI (Additional file 1: Table A1).

**Table 2:** Population characteristics of analyzed subjects.

	Total	Males	Females
% (*N*)	100 (1326)	51.1 (678)	48.9 (648)
	Mean (SD) or % (*n*)		
Age, years	15.2 (0.3)	15.2 (0.3)	15.2 (0.3)
Height*, cm	172 (8.2)	176 (7.4)	167 (6.0)
Study specific			
Study			
GINIplus control	38.3 (508)	38.2 (259)	38.4 (249)
GINIplus intervention	34.5 (457)	33.8 (229)	35.2 (228)
LISAplus	27.2 (361)	28.0 (190)	26.4 (171)
Study center			
Munich	63.3 (840)	63.1 (428)	63.6 (412)
Wesel	36.7 (486)	36.9 (250)	36.4 (236)
Early life events			
Parental atopy, yes	58.8 (780)	57.7 (391)	60.0 (389)
Parental education			
low (< 10 years of school)	5.2 (69)	5.8 (39)	4.6 (30)
medium (= 10 years of school)	26.7 (354)	27.0 (183)	26.4 (171)
high (> 10 years of school)	68.1 (903)	67.3 (456)	69.0 (447)
Maternal age at delivery >31 years*, yes	49.9 (662)	46.3 (314)	53.7 (348)
Maternal smoking during pregnancy, yes	12.0 (159)	11.9 (81)	12.0 (78)
Early second-hand smoke exposure at home (up to age 4), yes	32.7 (433)	32.6 (221)	32.7 (212)
Season of birth, winter	25.6 (339)	24.5 (166)	26.7 (173)
Birth weight*, g	3483 (442.0)	3541 (443.5)	3422 (432.6)
Exclusive breastfeeding >4 months, yes	60.6 (804)	58.3 (395)	63.1 (409)
Peak weight velocity*, kg/month	1.1 (0.2)	1.2 (0.2)	1.0 (0.2)
Peak height velocity*, cm/month	3.6 (0.4)	3.8 (0.4)	3.5 (0.4)
Lung infections (up to age 3)*, yes	31.2 (414)	34.8 (236)	27.5 (178)
Environmental and lifestyle factors at age 15			
Short-term air pollution			
NO_2_ (μg/m^3^)	20.4 (6.9)	20.5 (7.0)	20.3 (6.7)
PM_2.5_ mass (μg/m^3^)	14.7 (7.3)	14.4 (6.7)	15.0 (7.9)
PM_10_ mass (μg/m^3^)	18.9 (7.8)	18.6 (7.2)	19.3 (8.4)
Long-term air pollution			
NO_2_ (μg/m^3^)	21.2 (4.8)	21.2 (5.0)	21.2 (4.6)
PM_2.5_ mass (μg/m^3^)	14.8 (2.1)	14.8 (2.2)	14.8 (2.1)
PM_10_ mass (μg/m^3^)	22.1 (3.2)	22.0 (3.3)	22.1 (3.2)
Regular indoor second-hand smoke exposure^a^, yes	20.1 (266)	19.0 (129)	21.1 (137)
Active smoking, yes	5.4 (71)	5.6 (38)	5.1 (33)
Serum vitamin D^b^, nmol/l	68.4 (25.3)	67.5 (24.3)	69.3 (26.2)
Body mass index*, kg/m^2^	20.7 (3.0)	20.7 (3.2)	20.8 (2.8)
Allergic diseases at age 15			
Asthma, yes	6.3 (83)	7.4 (50)	5.1 (33)
Rhinitis, yes	18.9 (251)	20.9 (142)	16.8 (109)
Sensitization to			
Aeroallergens*, yes	45.4 (602)	51.5 (349)	39.0 (253)
Food allergens, yes	11.2 (148)	12.8 (87)	9.4 (61)
Spirometric parameters at age 15			
FVC, l*	4.08 (0.77)	4.50 (0.74)	3.64 (0.51)
FEV_1_, l*	3.52 (0.63)	3.83 (0.64)	3.19 (0.42)
FEV_1_/FVC, %*	86.7 (6.39)	85.3 (6.43)	88.1 (6.02)
PEF, l/s*	7.15 (1.28)	7.73 (1.3)	6.54 (0.93)
FEF_25_, l/s*	6.26 (1.17)	6.61 (1.27)	5.89 (0.92)
FEF_50_, l/s*	4.46 (1.05)	4.71 (1.14)	4.19 (0.87)
FEF_75_, l/s*	2.21 (0.72)	2.31 (0.78)	2.11 (0.63)
FEF_25–75_, l/s*	3.92 (0.92)	4.12 (1.01)	3.70 (0.77)

*Significant difference (*p*-value < 0.05) between males and females (t-test, Wilcoxon rank-sum test, or chi-square test)

^a^at least once a week or more. ^b^season-adjusted 25(OH)D concentration

FEV_1_: forced expiratory volume in 1 s. FVC: forced vital capacity. FEF_25_, FEF_50_, FEF_75_: forced expiratory flow rates at 25, 50 and 75% of exhaled FVC. FEF_25–75_: mean flow rate between 25 and 75% of FVC. PEF: peak expiratory flow. SD: standard deviation

Mean lung function parameters were higher among boys (*p* < 0.05), with the exception of FEV_1_/FVC, which was higher among girls (Table 2). Of 2358 participants with valid lung function measurements at the 15-year follow-up, 43.8% were excluded due to missing information on investigated factors. Lung function did not differ between the population analyzed and the other subjects with valid spirometry (Munich and Wesel) stratified by sex, with the exception that participating males had a slightly higher FEV_1_/FVC compared to non-participating males (Additional file 1: Table A2).

### Variable selection

Nearly all early life events and current environmental and lifestyle factors that showed significant associations with lung function in the selection models based on the total population remained in >70% of the models (Additional file 1: Tables A3 and A4). Exceptions included parental atopy (54% in FEV_1_/FVC model), parental education (52% in FEV_1_ model), and regular indoor second-hand smoke exposure at age 15 (66% in FEV_1_/FVC model, and slightly <70% in FEF_25–75_ model), which were less often included and therefore excluded from further analyses. Linear regression results for the included variables are shown in Table 3. Results considering secondary flow rates (PEF and FEF_25_ to FEF_75_) are shown in the additional file 1: Tables A4 and A5. The VIF was <2 for all variables in the final models, suggesting low multicollinearity.

**Table 3:** Coefficients (95%-confidence intervals) of regression models adjusted for covariates that remained stable in replication analyses

Spirometric parameter indicative of	Lung volume	Airways & volume	Airflow limitation	Airways
	FVC, ml	FEV_1_, ml	FEV_1_/FVC, %	FEF_25–75_, ml/s
Sex, male	347 (289, 405)	220 (167, 274)	−2.2 (−2.9,-1.4)	
Age, IQR years	47 (26, 69)	30 (11, 50)		
Height, IQR cm	637 (598, 676)	517 (481, 553)		508 (438, 577)
Early life events				
Peak weight velocity, IQR kg/month			−0.8 (−1.3, −0.3)	−88 (−155, −20)
Lung infections (up to age 3), yes		−55 (−102, −7)	−1.1 (−1.8,-0.3)	−159 (−258, −60)
Environment & lifestyle at age 15				
Regular indoor second-hand smoke exposure^a^, yes		−59 (−114, −5)		
Serum vitamin D^b^, IQR nmol/l	65 (34, 96)	32 (3, 61)	−0.6 (−1, −0.2)	
Body mass index, IQR kg/m^2^	222 (194, 250)	144 (118, 171)	−1.2 (−1.6,-0.8)	85 (31, 140)
Allergic diseases at age 15				
Asthma, yes	−118 (−216, −21)	−177 (−268, −86)	−1.8 (−3.1,-0.4)	−304 (−494, −115)
Study specific				
Study (LISAplus vs GINIplus)	68 (12, 124)			
Study center (Wesel vs Munich)	−66 (−119, −13)	−135 (−182, −88)	−1.6 (−2.3,-0.9)	−256 (−352, −161)

All associations were statistically significant (*p*-value < 0.05). Estimates for continuous variables are presented per interquartile range (IQR) increase (IQR: age (0.26 years), height (11 cm), peak weight velocity (0.28 kg/month), vitamin D (32.35 nmol/l), body mass index (3.56 kg/m^2^))

^a^at least once a week or more. ^b^season-adjusted 25(OH)D concentration. FEV_1_: forced expiratory volume in 1 s. FVC: forced vital capacity. FEF_25–75_: mean flow rate between 25 and 75% of FVC

### Individual factors - sex, age and height

Sex and height at lung function testing showed stable associations with all spirometric parameters, except for the association between FEF_25–75_ and sex and between FEV_1_/FVC and height (Table 3). Associations for age were found with FEV_1_ and FVC. Similar results were present considering further flow rates (Additional file 1: Table A5).

### Early life events

Lower lung function was associated with higher peak weight velocity and early lung infections (Table 3). Peak weight velocity was negatively associated with FEV_1_/FVC (β: -0.8%/IQR increase) and FEF_25–75_ (β: −88 ml/s/IQR increase). An impact of peak weight velocity on mainly the peripheral airways was also supported by the negative associations found with FEF_50_ and FEF_75_ (Additional file 1: Table A5). Early lung infections were also negatively associated with airway function as associations were found for FEV_1_ (β: −55 ml), FEV_1_/FVC (β: −1.1%) and FEF_25–75_ (β: −159 ml/s), but not for FVC. Furthermore, the larger and peripheral airways appeared to be affected by early lung infections as inferred from the negative associations seen with all flow rates (Additional file 1: Table A5).

While clear associations indicative of airway function and not for lung volume (FVC) were observed for peak weight velocity and early lung infections, no or only unstable associations were found for parental education, parental atopy, maternal age at delivery, maternal smoking during pregnancy, early second-hand smoke exposure at home, season of birth, birth weight, exclusive breastfeeding for at least four months and peak height velocity.

### Environmental and lifestyle factors at age 15

Regular indoor second-hand smoke exposure was negatively associated with FEV_1_ (β: −59 ml) (Table 3), as well as PEF and FEF_75_ (Additional file 1: Table A5), which supports the notion of a potential effect on the airways. Vitamin D concentrations were positively associated with FVC (β: 65 ml/IQR increase) and FEV_1_ (β: 32 ml/IQR increase), while the association with FEV_1_/FVC was negative (β: -0.6%/IQR increase). BMI was positively associated with FVC (β: 222 ml/IQR increase), FEV_1_ (β: 144 ml/IQR increase) and FEF_25–75_ (β: 85 ml/s/IQR increase) and negatively associated with FEV_1_/FVC (β: -1.2%/IQR increase). Positive associations between BMI and PEF, FEF_25_ and FEF_50_ were also found (Additional file 1: Table A5).

Spirometric parameters indicative of lung volumes were positively associated with vitamin D concentrations and BMI, while regular indoor second-hand smoke exposure showed some associations with flow rates, but no clear pattern. No associations were found with short-term or long-term air pollution exposure or active smoking at age 15 (prevalence for smoking 5.4%).

### Allergic diseases at age 15

Asthma was negatively associated with all lung function measures (Table 3), while no associations were found with current allergic rhinitis, or sensitization to food or aeroallergens. This was also true considering secondary parameters for airway function, except for PEF (Additional file 1: Table A5). Exclusion of subjects with asthma from the final models did not substantially modify the associations reported for early life events and environmental and lifestyle factors at age 15.

### Relative importance of factors in regression models

The total R^2^ was moderate in all models (R^2^ < 0.2), except for when FVC and FEV_1_ were the modelled outcomes, in which case it was higher (R^2^ = 0.68 and R^2^ = 0.59, respectively). As expected, the contribution to the total R^2^ of each model was highest for height (61.6–75.3%) and sex (23.9–27.2%) for almost all considered indices (Table 4, Additional file 1: Table A6). Due to the varying contribution of factors to different spirometric parameters, a direct comparison of all factors and parameters is not possible. The influence of early life events was primarily detectable for airway function. The highest contribution was found for FEV_1_/FVC with 22.4% and 7.2% for peak weight velocity and early lung infections, respectively. However, these two early life factors contributed less than 5% to the explained variance of the other lung function parameters of airway function (FEV_1_, FEF_25–75_). Among the current environmental and lifestyle factors, vitamin D concentrations (≤2.1%, maximum in FEV_1_/FVC) and regular indoor second-hand smoke exposure (0.2% in FEV_1_) contributed less than BMI, which contributed to almost all parameters (range 3.2% in FEF_25–75_ to 23.7% in FEV_1_/FVC).

**Table 4:**
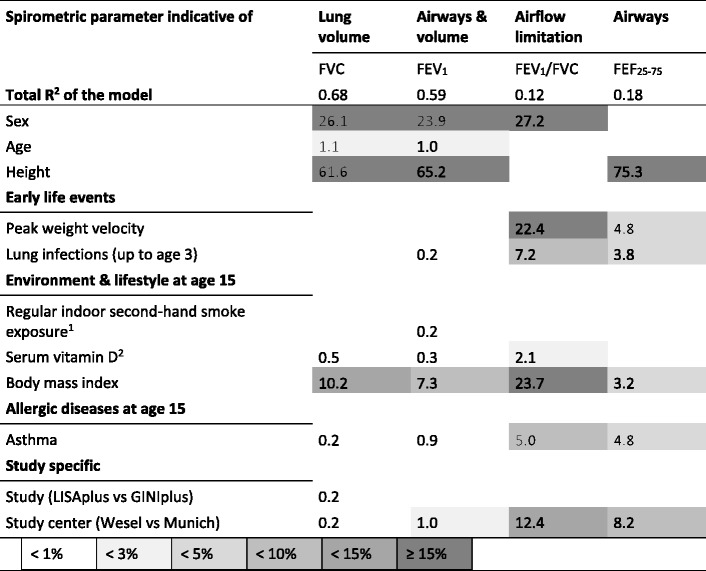
Relative importance of variables in final regression models (averaged *R* ^*2*^contribution)

Relative importance of variables in regression models adjusted for covariates that remained stable in replication analyses are displayed as normalized percent of *R* ^*2*^contribution averaged (unweighted) over variable orderings.

^1^at least once a week or more. ^2^season-adjusted 25(OH)D concentration. FEV_1_: forced expiratory volume in 1 s. FVC: forced vital capacity. FEF_25–75_: mean flow rate between 25 and 75% of FVC

## Discussion

Early life events, such as peak weight velocity and early lung infections, as well as current lifestyle factors, such as BMI, indoor second-hand smoke exposure and serum vitamin D concentrations, were associated with several spirometric parameters at age 15 years. The results of our study also confirm the well-established evidence supporting a role of sex, height and asthma status on lung function.

Among the early life events, a higher peak weight velocity was associated with airflow limitation and lower peripheral flow rates. Further, early lung infections were negatively associated with all lung function parameters except lung volume, suggesting that early lung infections may lead to low, long-term airflow limitation. A study by Svanes et al. reported an association of early childhood disadvantage factors, e.g. respiratory infections, maternal smoking and others, with lower lung function in adults and also a larger decline in lung function over time [6]. Early structural and functional changes on the developing or growing lung might lead to an impaired lung function and a higher susceptibility to lung diseases, but possible underlying mechanisms are not fully understood yet [1, 5, 6].

Regular indoor second-hand smoke exposure at age 15 years was associated with somewhat poorer airway function. The relative contribution of indoor second-hand smoke exposure was relatively low and no clear pattern with the lung function parameters could be determined. Higher vitamin D concentrations were primarily associated with volumetric indices, and with some airflow limitation, but with relatively small effects (<2.5% of R^2^). After height and sex, BMI contributed the most to the explained variance for nearly all spirometric parameters. BMI was positively associated with all lung function parameters indicative of lung volume and airway function, but also with airflow limitation. Similar associations have been reported in other studies among youth [15, 21].

Except for BMI, the relative importance of early life events and environmental and lifestyle factors was relatively low, in comparison to the contribution of sex and height. Nevertheless, early life events were primarily negatively associated with parameters indicative of airway function, while no associations were found for lung volume. This suggests that early life factors should be considered in studies focusing on airway function. Regular indoor second-hand smoke exposure at age 15, an environmental and lifestyle factor, showed the same tendency. On the contrary, positive associations were detected for vitamin D concentration with volumetric parameters and for BMI with both, volumes and airway function. Given the associations between higher weight gain in the first 2 years of life and current BMI with the spirometric indices, it appears that body weight at several points in life is important for lung function.

Associations of lung function with peak weight velocity [26, 27], lower respiratory infections [10, 14, 41], BMI [15, 21], vitamin D concentrations [16, 17] and second-hand smoke exposure [18, 20] have been also found in other studies. Only one previous study investigated associations between several early life events and current lifestyle factors with lung function [28]. This cross-sectional study investigated Tunisian children with an age range of 6–16 years (92 participants were 14–16 years old and would be comparable to our population). Similar to our results, associations between lung function with sex, height and weight were found. Strong associations with age were also reported [28], whereas the more narrow age distribution in our analysis was only associated with some spirometric parameters. As in our study, no associations between normal birth weight and lung function were observed in this previous publication [28]. Kotecha et al. reported an association between birth weight and lung function at age 8–9 years, but not at age 14–17 years, suggesting that the importance of early factors on lung function might differ by age [13].

As a complex framework of factors influences lung function [1, 2], we considered several early life and current environmental and lifestyle factors in adolescence to identify factors associated with lung function and their relative importance at age 15. Correlations, interactions or modulating effects between some investigated factors are very likely and study specific population characteristics might influence the impact and contribution of single factors. Study center was shown to be associated with lung function in our cohort but might partially stand as a surrogate for other factors not captured by our data. For example, there might be differences in lifestyle and environmental factors between the included rural (Wesel) and urban (Munich) areas.

Due to loss to follow-up, the prevalence of low education (<10 years of school) among the participants’ parents was only 5.2%, which may partially explain why socioeconomic status did not remain in the main models. In contrast to our results, a review covering different countries and age ranges showed that low socioeconomic status was associated with reduced lung function [42]. The underrepresentation of less educated families in our study might have also led to a lack of association with some factors that are correlated with socioeconomic status, such as maternal smoking during pregnancy (prevalence 12%), which otherwise, has been shown to have a negative effect in previous studies [18, 20]. Furthermore, results of a study among schoolchildren in Canada suggested a modifying effect of socioeconomic status on the association of air pollution and traffic exposure with respiratory symptoms and lung function [43]. In this study, a tendency for a higher risk of respiratory symptoms and lower lung function associated with traffic or air pollution exposure was seen in less educated households, although most associations were not statistically significant [43].

### Strengths and limitations

A major strength of this study is the investigation of a full range of standardized measured and visually inspected spirometric lung function parameters indicative of lung volume, as well as less often investigated lung function measures of larger conducting and peripheral airways, in two prospective birth cohorts. Furthermore, information on a broad range of early life events, environmental and lifestyle factors, and allergic diseases at age 15 was available for 1326 German adolescents, enabling this rarely applied comprehensive approach. Further factors that were available only in a subset of our population (986 subjects (74%)) e.g. having <2 or ≥2 siblings at age 15 (one variable for older, another for younger siblings), using gas for cooking and having mold at home, both asked in the first year of life, and daycare center attendance during the first three years of life would not have been included in the final models (based on best subset selection in this reduced population). This result and the limited data availability for these factors led to their exclusion from the main analysis. The inclusion of physical activity assessed by accelerometry would also have diminished our sample size to 721 subjects (54%) and was not shown to be associated with lung function in our cohort [35].

A major limitation of this study is selective loss to follow-up. For example, participants included in the analysis had higher parental education, more breastfeeding and less maternal smoking during pregnancy (Additional file 1: Table A1). Our results might therefore not be generalizable to all German adolescents. It is possible that covariates that were not associated with lung function in our population could play a role in others, pointing out a need for replication in other, larger studies. Further, study center (Munich/Wesel) was associated with lung function in our study population, which might suggest regional environmental and lifestyle differences not captured by the considered factors.

Our use of the Cp statistic as a selection criterion for automatic model selection might have resulted in the selection of a group of variables that would not have been selected using a different selection criterion (e.g. adjusted R^2^). To reduce model complexity, we chose to use the Cp statistic because it penalizes the number of included variables. Further, we replicated our analysis (*N* = 1000) in randomly chosen subpopulations to reduce potential selection bias attributable to influential cases.

## Conclusions

In addition to well-known measures included in lung function prediction equations (sex and height), as well as current asthma and BMI, our study showed that among a variety of factors considered in our analysis, weight gain and pulmonary infections during infancy were prevalent factors associated with lung function in 15-year-olds. While the early life factors were primarily associated with airway function, factors at age 15 showed associations with airway function as well as lung volume. Although our findings require replication in independent studies, they nevertheless highlight the need to include specific early life events and current lifestyle factors in studies on lung health among adolescents and suggest that effective health promotion should exist at all ages.

## Additional files

Additional file 1: Table A1. Population characteristics of analyzed participants in comparison to the initial study population for the Munich and Wesel study centers. **Table A2.** Characteristics of lung function parameters of analyzed participants in comparison to all other subjects with valid lung function measurements at age 15 in the Munich and Wesel study centers .**Table A3.** Coefficients (95% confidence intervals) of regression models with the lowest Mallows’ Cp, determined by best subset selection in the total population. **Table A4.** Distribution of the frequency of inclusion of each factor in 1000 replication analyses (%). **Table A5.** Coefficients (95% confidence intervals) of regression models of flow rates adjusted for covariates that remained stable in replication analyses. **Table A6.** Relative importance of variables in final regression models of flow rates (averaged R2 contribution). (PDF 548 kb)

## Abbreviations

BMI: Body mass index (kg/m^2^)
FEF_25_: Forced expiratory flow rate at 25% of exhaled FVC
FEF_25–75_: Mean flow rate between 25 and 75% of FVC
FEF_50_: Forced expiratory flow rate at 50% of exhaled FVC
FEF_75_: Forced expiratory flow rate at 75% of exhaled FVC
FEV_1_: Forced expiratory volume in 1 s
FVC: Forced vital capacity
GINIplus: German Infant study on the influence of Nutrition Intervention plus air pollution and genetics on allergy development
LISAplus: Life-style related factors on the development of the Immune System and Allergies in East and West Germany plus the influence of traffic emissions and genetics
NO_2_: Nitrogen dioxide
PEF: Peak expiratory flow
PM_10_: Particulate matter with diameters <10 μm
PM_2.5_: Particulate matter with diameters <2.5 μm
SD: Standard deviation. VIF: variance inflation factor
