# Supplementary material for: Which early life events or current environmental and lifestyle factors influence lung function in adolescents? – results from the GINIplus & LISAplus studies
Source: Respir Res. 2017 Jul 12;18:138. doi: 10.1186/s12931-017-0619-5 (PMC5508705; doi:10.1186/s12931-017-0619-5)
Supplement: Supplementary file 1 — Population characteristics of analyzed participants in comparison to the initial study population for the Munich and Wesel study centers. Table A2. Characteristics of lung function parameters of analyzed participants in comparison to all other subjects with valid lung function measurements at age 15 in the Munich and Wesel study centers .Table A3. Coefficients (95% confidence intervals) of regression models with the lowest Mallows’ Cp, determined by best subset selection in the total population. Table A4. Distribution of the frequency of inclusion of each factor in 1000 replication analyses (%). Table A5. Coefficients (95% confidence intervals) of regression models of flow rates adjusted for covariates that remained stable in replication analyses. Table A6. Relative importance of variables in final regression models of flow rates (averaged R2 contribution). (PDF 548 kb) [file 12931_2017_619_MOESM1_ESM.pdf]

# Additional file – Tables A1 to A6

**Table A1** Population characteristics of analyzed participants in comparison to the initial study population for the Munich and Wesel study centers

| | Study population analyzed (N=1326) | Study population excluded (N=6477)[A] | p-value* |
|---|---|---|---|
| | Mean (SD) or n/N (%) | | |
| Sex | | | |
| male | 678/1326 (51) | 3267/6316 (52) | 0.72 |
| female | 648/1326 (49) | 3049/6316 (48) | |
| Age at lung function measurement, years | 15.2 (0.3) | 15.3 (0.3) | < 0.01 |
| Height at lung function measurement, cm | 171.5 (8.2) | 171.6 (8.5) | 0.86 |
| **Study specific** | | | |
| Study | | | |
| GINIplus control | 508/1326 (38) | 3231/6477 (50) | < 0.01 |
| GINIplus intervention | 457/1326 (34) | 1795/6477 (28) | |
| LISAplus | 361/1326 (27) | 1451/6477 (22) | |
| Study center | | | |
| Munich | 840/1326 (63) | 3573/6477 (55) | < 0.01 |
| Wesel | 486/1326 (37) | 2904/6477 (45) | |
| **Early life events** | | | |
| Parental atopy, yes | 780/1326 (59) | 3194/6276 (51) | < 0.01 |
| Parental education | | | |
| low (< 10 years of school) | 69/1326 (5) | 827/6413 (13) | < 0.01 |
| medium ( = 10 years of school) | 354/1326 (27) | 1775/6413 (28) | |
| high (> 10 years of school) | 903/1326 (68) | 3811/6413 (59) | |
| Maternal age at delivery >31 years, yes | 662/1326 (50) | 2897/6461 (45) | < 0.01 |
| Maternal smoking during pregnancy, yes | 159/1326 (12) | 1069/5657 (19) | < 0.01 |
| Early second-hand smoke exposure at home (up to age 4), yes | 433/1326 (33) | 2117/4448 (48) | < 0.01 |
| Season of birth, winter | 339/1326 (26) | 1702/6477 (26) | 0.61 |
| Birth weight, g | 3482.7 (442.0) | 3463.7 (461.3) | 0.17 |
| Exclusive breastfeeding > 4 months, yes | 804/1326 (61) | 2498/4957 (50) | < 0.01 |
| Peak weight velocity, kg/month | 1.06 (0.22) | 1.09 (0.23) | < 0.01 |
| Peak height velocity, cm/month | 3.62 (0.42) | 3.67 (0.42) | < 0.01 |
| Lung infections (up to age 3), yes | 414/1326 (31) | 1371/4230 (32) | 0.44 |

| | Study population analyzed (N=1326) | Study population excluded (N=6477)[A] | p-value* |
|---|---|---|---|
| | Mean (SD) or n/N (%) | | |
| **Environmental and lifestyle factors at age 15** | | | |
| Short-term air pollution at lung function measurement | | | |
| $NO_2$ (µg/m$^3$) | 20.4 (6.9) | 20.4 (6.7) | 0.83 |
| $PM_{2.5}$ mass (µg/m$^3$) | 14.6 (7.3) | 15.7 (7.2) | < 0.01 |
| $PM_{10}$ mass (µg/m$^3$) | 18.9 (7.8) | 20.1 (7.8) | < 0.01 |
| Long-term air pollution at participant's home address | | | |
| $NO_2$ (µg/m$^3$) | 21.2 (4.8) | 22.0 (4.4) | < 0.01 |
| $PM_{2.5}$ mass (µg/m$^3$) | 14.8 (2.1) | 15.5 (2.2) | < 0.01 |
| $PM_{10}$ mass (µg/m$^3$) | 22.1 (3.2) | 22.9 (3.3) | < 0.01 |
| Regular indoor second-hand smoke exposure[1], yes | 266/1326 (20) | 438/2242 (20) | 0.74 |
| Active smoking, yes | 71/1326 (5) | 165/2246 (7) | 0.02 |
| Serum vitamin D concentration[2], nmol/l | 68.4 (25.3) | 66.3 (24.1) | 0.07 |
| Body mass index, kg/m$^2$ | 20.7 (3) | 21.2 (3.5) | < 0.01 |
| **Allergic diseases at age 15** | | | |
| Asthma, yes | 83/1326 (6) | 145/2938 (5) | 0.09 |
| Rhinitis, yes | 251/1326 (19) | 491/2839 (17) | 0.21 |
| Sensitization to | | | |
| Aeroallergens, yes | 602/1326 (45) | 560/1199 (47) | 0.54 |
| Food allergens, yes | 148/1326 (11) | 144/1199 (12) | 0.55 |

[A]Only participants from the Munich and Wesel study centers.*t-test, Wilcoxon rank-sum test or Pearson's chi-square test. [1]at least once a week or more. [2]season-adjusted 25(OH)D concentration.

**Table A2** Characteristics of lung function parameters of analyzed participants in comparison to all other subjects with valid lung function measurements at age 15 in the Munich and Wesel study centers

| | **Males** | | **Females** | |
|---|---|---|---|---|
| | **Study population analyzed (N=678)** | **Study population excluded (N=470)**[A] | **Study population analyzed (N=648)** | **Study population excluded (N=562)**[A] |
| **Spirometric parameter** | **Mean (SD)** | | **Mean (SD)** | |
| **FVC, l** | 4.50 (0.74) | 4.53 (0.77) | 3.64 (0.51) | 3.65 (0.49) |
| **$FEV_1$, l** | 3.83 (0.64) | 3.82 (0.67) | 3.19 (0.42) | 3.22 (0.42) |
| **$FEV_1$/FVC, %** | 85.33 (6.43)* | 84.46 (6.37) | 88.12 (6.02) | 88.39 (5.99) |
| **$FEF_{25-75}$, l/s** | 4.12 (1.01) | 4.03 (1.04) | 3.70 (0.77) | 3.77 (0.82) |
| **PEF, l/s** | 7.73 (1.3) | 7.64 (1.34) | 6.54 (0.93) | 6.55 (0.98) |
| **$FEF_{25}$, l/s** | 6.61 (1.27) | 6.46 (1.32) | 5.89 (0.92) | 5.92 (0.99) |
| **$FEF_{50}$, l/s** | 4.71 (1.14) | 4.58 (1.14) | 4.19 (0.87) | 4.29 (0.94) |
| **$FEF_{75}$, l/s** | 2.31 (0.78) | 2.26 (0.80) | 2.11 (0.63) | 2.15 (0.64) |

*Significant difference in lung function parameter between participants analyzed and those excluded, stratified by sex (t-test, $p<0.05$). [A]Participants with valid lung function measurements at age 15 from the Munich and Wesel study centers.

$FEV_1$: forced expiratory volume in 1 second. FVC: forced vital capacity. $FEF_{25}$, $FEF_{50}$, $FEF_{75}$: forced expiratory flow rates at 25, 50 and 75% of exhaled FVC. $FEF_{25-75}$: mean flow rate between 25 and 75% of FVC. PEF: peak expiratory flow. SD: standard deviation.

**Table A3** Coefficients (95% confidence intervals) of regression models with the lowest Mallows' Cp, determined by best subset selection in the total population

| **Spirometric parameter indicative of** | **Lung volume** | **Airways & volume** | **Airflow limitation** | **Airways** | **Function of larger conducting airways** | | **peripheral airways** | |
|---|---|---|---|---|---|---|---|---|
| | FVC, ml | $FEV_1$, ml | $FEV_1/FVC$, % | $FEF_{25-75}$, ml/s | PEF, ml/s | $FEF_{25}$, ml/s | $FEF_{50}$, ml/s | $FEF_{75}$, ml/s |
| Sex, male | **349 (291, 406)** | **217 (164, 270)** | **-2 (-2.8, -1.1)** | **118 (1, 234)** | **607 (473, 740)** | **274 (140, 409)** | **229 (99, 360)** | |
| Age, IQR years | **49 (27, 70)** | **34 (14, 54)** | | 31 (-10, 71) | 45 (-4, 93) | **72 (22, 123)** | 42 (-4, 88) | |
| Height, IQR cm | **646 (606, 687)** | **519 (483, 555)** | -0.4 (-1, 0.1) | **467 (389, 545)** | **709 (619, 799)** | **560 (469, 651)** | **482 (394, 571)** | **330 (274, 385)** |
| **Early life events** | | | | | | | | |
| Parental atopy, yes | | | **-0.7 (-1.4, -0.1)** | -77 (-172, 18) | **-124 (-238, -10)** | | | -67 (-142, 9) |
| Parental education# | | | | | | | | |
| high | | **-51 (-100, -2)** | | -90 (-193, 12) | **-154 (-282, -25)** | -118 (-243, 7) | -114 (-229, 2) | |
| low | | | | | -193 (-454, 67) | | | |
| Early second-hand smoke exposure at home (up to age 4), yes | | | | | | **133 (7, 258)** | | |
| Season of birth, winter | 46 (-8, 100) | | | | | | | |
| Birth weight, IQR g | -33 (-66, 1) | | | | | | | |
| Peak weight velocity, IQR kg/month | | | **-0.7 (-1.2, -0.2)** | **-110 (-180, -40)** | | | **-120 (-200, -41)** | **-89 (-143, -35)** |
| Lung infections (up to age 3), yes | | **-54 (-101, -6)** | **-1.0 (-1.7, -0.3)** | **-161 (-260, -62)** | **-131 (-249, -12)** | **-143 (-263, -23)** | **-169 (-281, -57)** | **-117 (-196, -38)** |
| **Environment & lifestyle at age 15** | | | | | | | | |
| Long term air pollution | | | | | | | | |
| $NO_2$, IQR µg/$m^3$ | | | -0.4 (-0.9, 0.1) | | | | | -43 (-98, 12) |
| $PM_{2.5}$ mass, IQR µg/$m^3$ | 86 (-29, 201) | | | | | | | |
| Regular indoor second-hand smoke exposure[1], yes | | **-65 (-120, -10)** | **-0.8 (-1.6, 0)** | **-132 (-246, -17)** | **-177 (-315, -40)** | -137 (-278, 5) | -124 (-254, 6) | **-126 (-217, -35)** |

| **Spirometric parameter indicative of** | **Lung volume** | **Airways & volume** | **Airflow limitation** | **Airways** | **Function of larger conducting airways** | | **peripheral airways** | |
|---|---|---|---|---|---|---|---|---|
| | FVC, ml | $FEV_1$, ml | $FEV_1$/FVC, % | $FEF_{25-75}$, ml/s | PEF, ml/s | $FEF_{25}$, ml/s | $FEF_{50}$, ml/s | $FEF_{75}$, ml/s |
| Serum vitamin D concentration[2], IQR nmol/L | **63 (32, 94)** | **31 (3, 60)** | **-0.6 (-1, -0.2)** | | | | | |
| Body mass index, IQR kg/m$^2$ | **226 (197, 254)** | **142 (116, 169)** | **-1.2 (-1.6, -0.8)** | **93 (38, 149)** | **261 (195, 327)** | **227 (161, 294)** | **149 (86, 211)** | |
| **Allergic diseases at age 15** | | | | | | | | |
| Asthma, yes | **-119 (-216, -22)** | **-178 (-269, -88)** | **-2 (-3.4, -0.6)** | **-346 (-538, -154)** | -198 (-434, 38) | **-398 (-627, -168)** | **-405 (-620, -190)** | **-193 (-346, -40)** |
| Rhinitis, yes | | | | | 119 (-28, 265) | | | |
| Aeroallergen sensitization, yes | | | 0.6 (-0.1, 1.3) | 78 (-17, 172) | | | | 70 (-5, 144) |
| **Study specific** | | | | | | | | |
| Study group# | | | | | | | | |
| GINIplus (intervention) | 49 (-7, 105) | | | | | | | |
| LISAplus | **97 (34, 160)** | 45 (-6, 97) | | | | 121 (-9, 252) | | |
| Study center, Wesel | **-145 (-276, -15)** | **-138 (-188, -87)** | **-1.3 (-2.1, -0.6)** | **-266 (-368, -164)** | **-325 (-446, -204)** | **-334 (-462, -206)** | **-282 (-396, -168)** | **-179 (-263, -95)** |

Significant associations are shown in bold ($p<0.05$). Estimates for continuous variables are presented per interquartile range (IQR) increase (IQR: age (0.26 years), height (11 cm), birth weight (587.5 g), peak weight velocity (0.28 kg/month), long term $NO_2$ (6.64 µg/m$^3$), long term PM 2.5 mass (4.00 µg/m$^3$), vitamin D concentration (32.35 nmol/l), body mass index (3.56 kg/m$^2$).

[#]Factor entered using dummy coding. [1]at least once a week or more. [2]season-adjusted 25(OH)D concentration.

$FEV_1$: forced expiratory volume in 1 second. FVC: forced vital capacity. $FEF_{25}$, $FEF_{50}$, $FEF_{75}$: forced expiratory flow rates at 25, 50 and 75% of exhaled FVC. $FEF_{25-75}$: mean flow rate between 25 and 75% of FVC. PEF: peak expiratory flow.

**Table A4** Distribution of the frequency of inclusion of each factor in 1000 replication analyses (%)

| **Spirometric parameter** | **FVC** | **$FEV_1$** | **$FEV_1$/FVC** | **$FEF_{25-75}$** | **PEF** | **$FEF_{25}$** | **$FEF_{50}$** | **$FEF_{75}$** |
|---|---|---|---|---|---|---|---|---|
| Sex, male | 100 | 100 | 100 | 62 | 100 | 100 | 98 | 2 |
| Age, years | 100 | 98 | 14 | 35 | 51 | 90 | 54 | 23 |
| Height, cm | 100 | 100 | 42 | 100 | 100 | 100 | 100 | 100 |
| **Early life events** | | | | | | | | |
| Parental atopy, yes | 10 | 26 | 54 | 38 | 72 | 11 | 31 | 36 |
| Parental education[#] | | | | | | | | |
| high | 12 | 52 | 20 | 50 | 57 | 52 | 49 | 34 |
| low | 15 | 10 | 3 | 3 | 31 | 8 | 8 | 3 |
| Maternal age at delivery >31 years, yes | 7 | 3 | 20 | 17 | 12 | 8 | 13 | 16 |
| Maternal smoking during pregnancy, yes | 29 | 23 | 3 | 1 | 5 | 6 | 3 | 1 |
| Early second-hand smoke exposure at home (up to age 4), yes | 16 | 10 | 2 | 1 | 31 | 66 | 2 | 1 |
| Season of birth, winter | 43 | 18 | 1 | 2 | 10 | 9 | 2 | 10 |
| Birth weight, g | 44 | 8 | 5 | 4 | 18 | 11 | 20 | 10 |
| Exclusive breastfeeding > 4 months, no | 3 | 2 | 2 | 3 | 19 | 4 | 6 | 2 |
| Peak weight velocity, kg/month | 3 | 22 | 93 | 92 | 1 | 25 | 95 | 97 |
| Peak height velocity, cm/month | 23 | 18 | 3 | 4 | 12 | 3 | 4 | 11 |
| Lung infections (up to age 3), yes | 6 | 73 | 94 | 98 | 74 | 80 | 95 | 95 |
| **Environment & lifestyle at age 15** | | | | | | | | |
| Short-term air pollution | | | | | | | | |
| $NO_2$ (µg/$m^3$) | 2 | 4 | 17 | 24 | 7 | 4 | 41 | 13 |
| $PM_{2.5}$ mass (µg/$m^3$) | 4 | 4 | 11 | 9 | 4 | 3 | 12 | 3 |
| $PM_{10}$ mass (µg/$m^3$) | 9 | 1 | 39 | 12 | 3 | 2 | 17 | 10 |

| Spirometric parameter | FVC | $FEV_1$ | $FEV_1$/FVC | $FEF_{25-75}$ | PEF | $FEF_{25}$ | $FEF_{50}$ | $FEF_{75}$ |
|---|---|---|---|---|---|---|---|---|
| Long-term air pollution | | | | | | | | |
| $NO_2$ (µg/m$^3$) | 6 | 19 | 31 | 27 | 19 | 6 | 28 | 24 |
| $PM_{2.5}$ mass (µg/m$^3$) | 36 | 31 | 24 | 10 | 21 | 8 | 10 | 9 |
| $PM_{10}$ mass (µg/m$^3$) | 8 | 4 | 8 | 9 | 16 | 3 | 13 | 20 |
| Regular indoor second-hand smoke exposure[1], yes | 21 | 80 | 66 | 70 | 86 | 54 | 51 | 95 |
| Active smoking, yes | 9 | 12 | 7 | 7 | 21 | 8 | 6 | 9 |
| Serum vitamin D concentration [2], nmol/l | 100 | 74 | 85 | 1 | 16 | 2 | 4 | 32 |
| Body mass index, kg/m$^2$ | 100 | 100 | 100 | 97 | 100 | 100 | 100 | 8 |
| **Allergic diseases at age 15** | | | | | | | | |
| Asthma, yes | 79 | 99 | 84 | 97 | 39 | 97 | 99 | 79 |
| Rhinitis, yes | 7 | 5 | 6 | 8 | 31 | 3 | 10 | 5 |
| Sensitization to | | | | | | | | |
| Aeroallergens, yes | 1 | 11 | 41 | 44 | 4 | 2 | 32 | 45 |
| Food allergens, yes | 3 | 2 | 4 | 4 | 3 | 8 | 5 | 2 |
| **Study specific** | | | | | | | | |
| Study group[#] | | | | | | | | |
| GINIplus (intervention) | 53 | 17 | 7 | 6 | 13 | 6 | 3 | 4 |
| LISAplus | 90 | 54 | 16 | 3 | 4 | 53 | 7 | 10 |
| Study center, Wesel | 82 | 99 | 63 | 86 | 99 | 99 | 83 | 78 |

Factors that were included in >70% of replication analyses are shaded in gray.

[#]Factor entered using dummy coding. [1]at least once a week or more. [2]season-adjusted 25(OH)D concentration. $FEV_1$: forced expiratory volume in 1 second. FVC: forced vital capacity. $FEF_{25}$, $FEF_{50}$, $FEF_{75}$: forced expiratory flow rates at 25, 50 and 75% of exhaled FVC. $FEF_{25-75}$: mean flow rate between 25 and 75% of FVC. PEF: peak expiratory flow.

**Table A5** Coefficients (95% confidence intervals) of regression models of flow rates adjusted for covariates that remained stable in replication analyses

| Spirometric parameter indicative of airway function of | larger conducting airways | | peripheral airways | |
|---|---|---|---|---|
| | PEF, ml/s | $FEF_{25}$, ml/s | $FEF_{50}$, ml/s | $FEF_{75}$, ml/s |
| Sex, male | 602 (470, 735) | 285 (150, 420) | 227 (97, 358) | |
| Age, IQR years | | 63 (14, 112) | | |
| Height, IQR cm | 713 (623, 803) | 555 (464, 646) | 488 (399, 576) | 328 (272, 383) |
| **Early life events** | | | | |
| Parental atopy, yes | -123 (-236, -11) | | | |
| Peak weight velocity, IQR kg/month | | | -122 (-202, -43) | -82 (-136, -28) |
| Lung infections (up to age 3), yes | -140 (-258, -23) | -150 (-270, -29) | -171 (-283, -58) | -118 (-198, -39) |
| **Environment & lifestyle at age 15** | | | | |
| Regular indoor second-hand smoke exposure[1], yes | -164 (-301, -27) | | | -130 (-220, -39) |
| Serum vitamin D concentration[2], IQR nmol/l | | | | |
| Body mass index, IQR kg/m$^2$ | 265 (200, 331) | 231 (165, 297) | 149 (87, 212) | |
| **Allergic diseases at age 15** | | | | |
| Asthma, yes | | -391 (-621,-161) | -392 (-607,-177) | -171 (-323, -20) |
| **Study specific** | | | | |
| Study center (Wesel vs Munich) | -314 (-430,-198) | -303 (-419,-186) | -266 (-375,-157) | -201 (-277,-125) |

All associations were statistically significant ($p<0.05$). Estimates for continuous variables are presented per interquartile range (IQR) increase (IQR: age (0.26 years), height (11 cm), peak weight velocity (0.28 kg/month), vitamin D (32.35 nmol/l), body mass index (3.56 kg/m$^2$)).

[1]at least once a week or more. [2]season-adjusted 25(OH)D concentration.

$FEF_{25}$, $FEF_{50}$, $FEF_{75}$: forced expiratory flow rates at 25, 50 and 75% of exhaled forced vital capacity. PEF: peak expiratory flow.

**Table A6** Relative importance of variables in final regression models of flow rates (averaged $R^2$ contribution)

| Spirometric parameter indicative of airway function of | larger conducting airways | | peripheral airways | |
|---|---|---|---|---|
| | PEF | $FEF_{25}$ | $FEF_{50}$ | $FEF_{75}$ |
| **Total $R^2$ of the model** | 0.38 | 0.24 | 0.18 | 0.13 |
| Sex | 32.9 | 21.8 | 18.2 | |
| Age | | 2.6 | | |
| Height | 55.0 | 54.8 | 55.5 | 68.3 |
| **Early life events** | | | | |
| Parental atopy | 0.7 | | | |
| Peak weight velocity | | | 4.0 | 4.1 |
| Lung infections (up to age 3) | 0.4 | 1.3 | 3.3 | 5.1 |
| **Environment & lifestyle at age 15** | | | | |
| Regular indoor second-hand smoke exposure[1] | 0.6 | | | 5.1 |
| Serum vitamin D concentration[2] | | | | |
| Body mass index | 8.2 | 12.1 | 6.9 | |
| **Allergic diseases at age 15** | | | | |
| Asthma | | 3.0 | 5.5 | 3.9 |
| **Study specific** | | | | |
| Study center (Wesel vs Munich) | 2.1 | 4.4 | 6.7 | 13.5 |

| < 1% | < 3% | < 5% | < 10% | < 15% | ≥ 15% |
|---|---|---|---|---|---|

Relative importance of variables in regression models adjusted for covariates that remained stable in replication analyses are displayed as normalized percent of $R^2$contribution averaged (unweighted) over variable orderings.

[1]at least once a week or more. [2]season-adjusted 25(OH)D concentration.

$FEF_{25}$, $FEF_{50}$, $FEF_{75}$: forced expiratory flow rates at 25, 50 and 75% of exhaled forced vital capacity. PEF: peak expiratory flow.
